# P02.09. An open label pilot study of homeopathic treatment of Attention Deficit Hyperactivity Disorder (ADHD)

**DOI:** 10.1186/1472-6882-12-S1-P65

**Published:** 2012-06-12

**Authors:** D Brulé, H Boon

**Affiliations:** 1 Riverdale Homeopathic Clinic, Toronto, Canada; 2 University of Toronto, Toronto, Canada

## Purpose

The objectives of this open label pilot study of the homeopathic treatment of Attention Deficit Hyperactivity Disorder (ADHD) are to: (1) Develop preliminary estimates of treatment effects including magnitude, direction, variability and standard deviation in order to determine potential for future study; (2) Determine the length of time and number of remedies needed to achieve a 25% reduction in ADHD symptoms as measured using the Conners 3 Parent questionnaire; (3) Assess the feasibility of the recruitment plan and the outcome measure schedules.

## Methods

Participants age 6-16 with ADHD (confirmed by the study psychiatrist) of any subtype were invited to participate if they were on a stable medication dose for a minimum of six weeks. Participants with severe psychiatric co-morbidities were excluded. Participants received 10 homeopathic consultations from one of two experienced homeopaths over 9 months who prescribed single homeopathic remedies on an individualized basis from a list of 115 potential substances. Remedies, potencies, and dosing frequencies could be changed at any of the appointments. Outcomes, including Conners 3 Parent questionnaire, were measured at baseline and at each follow-up appointment.

## Results

Thirty-six participants (including six females) were recruited over an 11-month period with a mean age of 9.5 (range 6-16). Two participants have withdrawn to date. Of the 15 participants who have completed 8 or more consultations, 11 (73%) have had at least a 25% reduction in their symptoms. Of those participants who have had a 25% reduction, the average time to reach that threshold was 2.6 months and they tried on average 2.2 remedies. Data collection for the majority of the participants will be complete by May 2012.

## Conclusion

Preliminary findings from this open-label pilot study indicate that proceeding with the design and conduct of a randomized controlled trial investigating the effects of homeopathic treatment of ADHD is warranted.

